# Somatic Cell Count in Goat Milk: An Indirect Quality Indicator

**DOI:** 10.3390/foods10051046

**Published:** 2021-05-11

**Authors:** Klára Podhorecká, Markéta Borková, Miloslav Šulc, Růžena Seydlová, Hedvika Dragounová, Martina Švejcarová, Jitka Peroutková, Ondřej Elich

**Affiliations:** 1 Department of Chemistry, Faculty of Agrobiology, Food and Natural Resources, Czech University of Life Sciences Prague, Kamýcká 129, 16500 Prague, Czech Republic; 2 Dairy Research Institute, Ke Dvoru 12a, 16000 Prague, Czech Republic; borkova@milcom-as.cz (M.B.); seydlova@milcom-as.cz (R.S.); dragounova@milcom-as.cz (H.D.); svejcarova@milcom-as.cz (M.Š.); peroutkova@milcom-as.cz (J.P.); elich@milcom-as.cz (O.E.); 3 Food Research Institute Prague, Radiova 1285, 10200 Prague, Czech Republic; miloslav.sulc@vupp.cz

**Keywords:** goat milk, somatic cells, mastitis-causing bacteria (MCB), *Staphylococcus* spp., milk quality

## Abstract

A high somatic cell count (SCC) impacts dairy quality to a large extent. The goal of this work was to investigate differences in goat milk composition and technological parameters according to SCC cut-off (600, 700, 800, and 1000.10^3^/mL). Thirty-four individual milk samples of White Shorthair goats in a similar stage of lactation were investigated. The first differences in milk quality appeared already at SCC cut-off of 600.10^3^/mL (5.58 LSCS-linear somatic cell score), yet the most striking differences were found for SCC over 1000.10^3^/mL (6.32 LSCS), which was expressed by lowering heat stability (126 vs. 217 s, *p* = 0.034), increasing protein (3.41 vs. 3.04%, *p* = 0.009), casein (2.80 vs. 2.44%, *p* = 0.034) and chloride (164 vs. 147 mg/100 mL, *p* = 0.004) levels, as well as non-fat dry matter (8.79 vs. 8.45%, *p* = 0.045). It has been shown that low levels of *Staphylococcus* spp. bacteria (120–1600 CFU/mL) in the mammary gland correlated with decreased lactose content (4.60 vs. 4.47 g/100 g, *p* = 0.022). Since our results indicate that even low SCC values may significantly affect the technological properties of goat milk, SCC should therefore be routinely screened and reported to dairy manufacturers to assure the consumer of high end-product quality.

## 1. Introduction

Somatic cell count (SCC) is considered an indirect indicator of the milk’s hygienic quality. Higher SCC values above a typical physiological range indicate microbial inflammation of the mammary gland. This is not true for goat milk (an apocrine type of milk), which naturally contains higher amounts of epithelium cells and their fragments, arising from epithelial desquamation and physiological regeneration of mammary alveoli [1,2]. For this reason, raw goat milk typically has a higher SCC compared to cow milk.

Somatic cells are a natural component of milk, and they comprise epithelial cells (as mentioned above) and leukocytes (white blood cells) [3]. Leukocytes play an important role in an animal’s immune response, their main task being to protect the body against foreign invaders (e.g., pathogenic bacteria) [2]. There are many types of leukocytes found in milk, such as macrophages, lymphocytes, and polymorphonuclear leukocytes (PMN), the ratios of which differ by animal species. PMN are the main leukocyte type found in the milk of healthy goats [4,5] compared to cow and sheep milk, which predominantly contains macrophages [3]. Mastitis increases PMN content in cow and sheep milk, but not in goat milk, in which they still represent the dominant type [4,5]. 

Enzymes in milk are of endogenous origin (originating from somatic cells, blood plasma, or fat globule membranes) or exogenous origin (microbial enzymes). Somatic cells are a source of a variety of enzymes which characterize each type of leukocyte. Macrophages and PMN (the most abundant leukocytes in the milk of healthy cows and goats) are an important source of proteases such as lysosomal cathepsin and elastase enzymes [6]. 

Plasmin is the major proteolytic enzyme in milk [7], and it is commonly found in milk produced by both healthy and diseased mammary glands [8]. It is formed from its zymogen (plasminogen) catalyzed by plasminogen activator protein, which is likely produced by somatic cells such as macrophages [7]. Plasmin synthesis and function is a good example how somatic cells in milk might directly affect the composition and technological parameters of goat milk. To date we still don’t have a complete picture of the role of enzymes in milk (e.g., plasmin and other endogenous enzymes produced by somatic cells) and the factors that contribute to their activity. It is likely that these enzymes cause postsecretional changes in milk composition and impact its quality, as well as its technological parameters.

Raynal-Ljutovic et al. [9] have shown that somatic cells exert proteolytic activity that alters casein fractions, which, in turn, impact technological properties (e.g., lowering curd firmness). Endogenous enzymes found in somatic cells may even influence organoleptic properties of milk [10].

Typically, goat milk contains high SCC levels on average (e.g., 1100 resp. 1300.10^3^/mL) as found by Bogdanovičová et al. [11] and Novotná et al. [12]. Such values are alarming, and it is necessary to investigate the causes for these high values and how they impact goat milk quality. High SCC values found in milk from healthy (mastitis-free) goats should therefore be a clear signal as to whether such milk is suitable for technological processing in a dairy plant. The goal of this research was to shed some light on how different SCC levels (600, 700, 800, and 1000.10^3^/mL) affect the qualitative, quantitative, and technological properties of milk from healthy goats. 

## 2. Materials and Methods

### 2.1. Chemicals

Chymosin enzyme (Chr. Hansen, Avedore, Denmark); ammonium thiocyanate solution, silver nitrate (Lach-ner, s.r.o., Neratovice, Czech Republic); ammonium iron (III) sulfate dodecahydrate ACS grade (VWR International, s.r.o., Stříbrná Skalice, Czech Republic); nitric acid 67%, Analpure grade (Analytika^®^, spol. s.r.o., Prague, Czech Republic) was purchased.

### 2.2. Farm and Animals

One of the largest organically operated goat farms in the Czech Republic (500 heads in 2020), which enabled a sufficient number of animals of the same breed and kept in the same conditions to be obtained, was selected for the study. In total, 34 White Shorthair goats in their second or third lactation (parturitions between 7–27 March 2020), housed in the same environment and fed with uniformed ration (consisting of fed hay, haylage, pasture, and granulated concentrated feed), were included in the study.

### 2.3. Milk Sampling, Milking 

Thirty-four individual milk samples were taken by manual milking between 18–25 August 2020. The milking was carried out by the farm personnel in disposable sterile gloves in the following way: the udder was washed, the teats wiped with a single-use disinfecting wipe, the first couple of jets were discarded, the further jets were collected separately into sterile bottles (for MCB, TMC), and manual milking ensued. The milk samples were cooled down to 4–6 °C and immediately transported to the laboratories for further analyses. 

### 2.4. Testing Laboratories

Microbiological testing was performed by Dairy Research Institute (Prague, Czech Republic) and veterinary laboratory VEDIA (Strakonice, Czech Republic); chemical and technological testing was performed by Dairy Research Institute (Prague, Czech Republic) and Czech University of Life Sciences (Prague, Czech Republic).

### 2.5. Methods

#### 2.5.1. Somatic Cell Count (SCC)

SCC was measured in cassettes by DeLaval Cell Counter (DeLaval, Stockholm, Sweden) on site directly after milking. The SCC values were transformed into a linear somatic cell score (LSCS) using the conversion LSCS = 3 + log_2_ (SCC/100), according to Wiggans and Shook (1987) [13], in order to normalize the data.

#### 2.5.2. Total Microbial Count (TMC), Identification of Bacteria and Their Count

TMC was quantified using GTK-M agar plates (MILCOM a.s., Tábor, Czech Republic) by culturing at 30 °C for 72 h under the aerobic conditions, according to ČSN EN ISO 4833-1 [14], which is the Czech equivalent of ISO 4833-1.

Identification of bacteria and their count: pathogenic bacteria in milk were determined by the Veterinary Laboratory Vedia s.r.o. (Strakonice, Czech Republic). The presence of coliform bacteria, staphylococci, streptococci, and corynebacteria was monitored. Basic cultivation of bacteria occurred on the Columbia blood agar (Oxoid, UK). ENDO agar (Oxoid, UK) was used to cultivate coliform bacteria. Enterococcus Selective Agar-BAA (Oxoid, UK) was used to distinguish streptococci and enterococci, and Edwards agar (Oxoid, UK) was used for streptococcal culture, and for staphylococci, Baird-Parker agar (Oxoid, UK) and Staphylococcus agar (Sigma-Aldrich, USA) was applied. A coagulase test was used to distinguish between coagulase-negative and -positive staphylococci (Staphylase Test, Oxoid, UK). 

When necessary, a microflex LT MALDI TOF mass spectrometer (Bruker Daltonics, Bremen, Germany) with a microSCOUT ion source and a TOF flight time analyzer (Bruker Daltonics, Bremen, Germany) in conjunction with the MALDI Biotyper software system (Bruker Daltonics, Bremen, Germany) was used to identify bacterial species.

#### 2.5.3. Rennetability (Chymosin-Induced Coagulation)

Milk rennetability was measured, in line with Desobry-Banon et al. [15], as time needed for the milk to start curdling after chymosin enzyme addition. One m of freshly prepared chymosin solution (equivalent to curdling activity 100,000) was added to 100 mL of milk kept at 35 °C, gently swirled, and the appearance of the first curds was observed. 

#### 2.5.4. Heat Stability (Thermostability)

A modified method by Robitaille and Ayers [16] was used in which the heat stability is measured as time needed for the milk to begin curdling by heat treatment. 2.5 mL of milk was pipetted into a glass test tube (Pyrex test tubes 10 × 0.75 cm) and stoppered with a silicone rubber. The test tubes were immersed in a thermostatically controlled oil bath heated to 140 °C, and the appearance of the first curds was observed. 

#### 2.5.5. pH

The pH was measured by a glass electrode using SensION+ pH 1 instrument (Hach, Prague, Czech Republic) calibrated daily with buffers pH 4.00 and 7.00.

#### 2.5.6. Fat, Protein, Lactose, Casein, Non-Fat and Total Dry Matter, Chlorides 

The milk was analyzed on DairySpec FT (Bentley Instruments) that was calibrated with goat milk samples using the following reference methods: fat (ČSN EN ISO 1211 [17], the Czech equivalent of ISO 1211), protein (ČSN EN ISO 8968-1 [18], the Czech equivalent of ISO 8968-1), casein (ČSN ISO 17997-1 [19], the Czech equivalent of ISO 17997-1), lactose (IDF 79-2) [20], non-fat and total dry matter (ČSN ISO 6731 [21], the Czech equivalent of ISO 6731).

Chlorides were quantified by argentometry (in three replicates), using 0.1 M ammonium thiocyanate solution in line with ČSN 57 0530 [22]. To 10 g of milk sample, 5 mL of 25% of nitric acid and 1 mL of saturated ammonium iron (III) sulfate solution (indicator) was added and carefully mixed, followed by 10 mL of 0.1 M silver nitrate and titrated.

### 2.6. Statistics

Statistical analyses were performed by Dell^TM^Statistica^TM^13.1 software (Dell Inc., Round Rock, TX, USA). The non-parametric data in our study were analyzed using the Kruskal-Wallis test for independent variables (*p* < 0.05). For more in-depth statistical details, Dunn’s multiple comparison test was carried out. The descriptive statistics use medians; however, for better comparison of results in tables, means and standard deviations (SD) are also used. 

## 3. Results and Discussion

Goat milk quality and composition is influenced by many factors, such as breed type, feeding, lactation stage, lactation number, kidding date, and overall health condition [23,24]. In this experiment, we tried to reduce the influence of these factors on how SCC affects milk quality and composition. Therefore, our animals were housed at the same farm, were fed the same diet, had the same kidding date (7–27 March 2020) and were in the same lactation stage. Only animals with no obvious udder or teat problems (redness, etc.) were included in the experiment. Twelve milk samples came from goats on the second lactation and 22 samples from goats on the third lactation. Because no statistically significant differences in any milk parameter (see Table 1) regarding the lactation number were found, the influence of the lactation number was further disregarded, and thus we were able to work with a larger set of data (34 in total). Nevertheless, this dataset is not extensive due to the very careful selection and correction for factors such as breeding environment, kidding date, lactation phase, etc.

Milk quality is affected by bacteria [25]. Therefore, the herd was screened for mastitis-causing bacteria (MCB), which has oftentimes been neglected in other studies investigating SCC (linear somatic cell score (LSCS), resp.) in relation to milk quality. MCB were detected in the range of 120 to 1600 CFU/mL in 11 milk samples (32.4%), most often as *Staphylococcus PK-*(delta hemolysin negative), rarely as *Staphylococcus PK-*(delta hemolysin positive) and only in two cases as *Staphylococcus aureus* (120 and 340 CFU/mL, resp.). Both *S. aureus* samples were within the allowed legislative requirements for raw goat milk (*S. aureus* < 500 CFU/mL and TMC < 500 × 10^3^/mL), and thus could have otherwise been used for dairy manufacturing. On the other hand, for pasteurized goat milk, there are no limits set by the legislation for *S. aureus*, yet TMC must be < 1500 × 10^3^ CFU/mL [26]. In our samples, TMC did not exceed 7300 CFU/mL (mean = 633 CFU/mL), thus no significant microbial colonization of the mammary gland was revealed, and all milk samples were of a very good hygienic standard. 

Table 2 presents results grouped according to detected SCC (LSCS, resp.) and MCB. *Staphylococcus* spp. distribution (disregarding SCC) is similar in L and H group (27.3–35.3%, Table 3). Higher SCC levels in our study might have been caused by factors other than MCB, e.g., the animal’s physiology, genotype [27], or by other non-infectious factors such as estrus, seasonal influences, or parity [28,29]. External stresses experienced by the animal can also influence milk SCC [2,29]. The even distribution of *Staphylococcus* spp. bacteria (MCB) in our samples might have been caused by the above *Staphylococcus* types as well as by their actual low counts. Leitner et al. [25] and Bagnicka et al. [5] showed that coagulase-negative *Staphylococci* are the most often detected MCB with subclinical manifestation, which might not express itself in an elevated SCC. A different situation can be observed in the presence of *S. aureus* in milk, which always raises SCC levels [5,30]. This is in line with our observation of the two *S. aureus* positive milk samples which had an SCC above 1000.10^3^/mL (6.32 LSCS).

Only milk samples with a *Staph.* count up to 100 CFU/mL were included in further SCC analysis (samples with *Staph.* count > 100 CFU/mL were excluded due to the excessive presence of MCB). Therefore, it can be assumed that samples with a low *Staph.* count (<100 CFU/mL) came from goats with healthy udders (not even affected by subclinical mastitis). Subclinical mastitis is rather hard to detect directly on a farm since it is not visually manifested by redness, swelling, fever, loss of appetite, or fatigue [31]. Twenty-three (out of the 34) milk samples were nearly mastitis-free (Table 2). These 23 samples were further subdivided into two groups with a lower (LZ) or higher (HZ) SCC than the particular SCC cut-off (600, 700, 800, or 1000.10^3^/mL); for instance, SCC < 600.10^3^/mL and SCC > 600.10^3^/mL. SCC cut-off 900 doesn’t exist because no investigated milk sample contained SCC 900–999.10^3^/mL. Each SCC cut-off group (low vs. high) was investigated for differences in the milk’s chemical composition and technological parameters. Our results show that SCC influences milk yield, milk composition, and technological parameters (Table 4). The most striking differences were found for an SCC over 1000.10^3^/mL (6.32 LSCS), yet some differences appeared already in samples with an SCC over 600.10^3^/mL (5.58 LSCS, resp.) (milk yield, chlorides). From our results, any HZ group tended toward higher chloride content. Chloride ions (in the form of NaCl) contribute to milk’s osmotic pressure. Higher NaCl levels found in milk, however, negatively impact its technological parameters, such as rennetability (coagulation) and curd firmness [32].

With increasing SCC, the differences between LZ and HZ groups were more significant. In the HZ group of SCC 700.10^3^/mL (5.81 LSCS), we could observe increased protein and chloride contents, while lactose content decreased. A similar situation is seen in the group SCC 800.10^3^/mL, where the heat stability starts to shorten. The most significant differences (between HZ and LZ groups) were found at SCC 1000.10^3^/mL (6.32 LSCS), at which the milk contained more non-fat dry matter (NFDM) caused by higher casein and protein contents. Moreover, the HZ group of samples (SCC 1000.10^3^/mL) also had increased chloride levels. According to the literature, the relation between SCC and goat milk composition is still inconclusive [9,32], which might have been caused by insufficient correction for factors influencing milk composition. Leitner et al. [25], Bernacka [33], and Barrón-Bravo et al. [34] all found a positive correlation between SCC and protein content (caused by increased whey protein content). Our observations that SCC increases protein and casein contents are in line with data published by Stocco et al. [32].

Increased casein content in HZ (*Staph.* count <100 CFU/mL and SCC >1000.10^3^/mL) could first appear as convenient, yet, in fact, result in inconvenient technological parameters such as heat stability and rennetability. This might have been caused by factors other than chlorides. It is important to underscore that somatic cells have their own enzymes which might influence the technological parameters of milk. Thus, increased milk SCC might lead to higher enzyme and enzyme activator/inhibitor content. In this respect Bagnicka et al. [5] as well as Alhussien et al. [3] found that PMN produce a variety of enzymes-cathepsin (B, C, D, L, G, S), elastase, lipoprotein lipase, and collagenase [6]. 

One of the most studied endogenous enzymes in milk is cathepsin D (a lysosomal aspartic endopeptidase), the activity of which is correlated with SCC [7,35]. α_S1_-, α_S2_-, β- and κ-casein are all hydrolyzed by its active form [10]. Higher α_S1_- and α_S2_-casein contents do increase coagulation time and curd firmness [36], whereas their (proteolytic) fragments might worsen the milk’s technological parameters [8,25]. Our ongoing research aims to investigate how SCC influences casein content, casein fractions, and actual fragments being formed. From the information above, it is evident that the intricacies of endogenous enzymes (originating in somatic cells) are enormously complex. In 2013, Janjanam et al. [37] studied proteolytic fragments of epithelial cells in cow milk and identified 497 unique proteins (409 were found in the databases), and 18% were classified as enzymes. It is more than obvious that there is a need to not only investigate the relationship between SCC and goat milk quality, but also to pay closer attention to somatic cell differentiation and to the endogenous enzymes.

The preceding paragraphs (and Table 4) tried to explain the effects SCC had on milk quality in samples which were almost MCB-free (*Staph.* count <100 CFU/mL). We tried to describe those samples in great detail, since we feel that other studies devoted to SCC and goat milk quality do not sufficiently pay attention to somatic cells (from healthy udders) and bacteria (MCB). The results from these experiments do not sufficiently consider the possible colonization of the mammary gland by MCB. This is why we analyzed the SCC and milk quality in relation to MCB (*Staphylococcus* spp.), even though their contents were rather low (Table 5). In our evaluation, we divided the 34 milk samples into groups Z (*Staph.* count <100/mL) and B (*Staph.* count >100/mL) and found no relationship to SCC (LSCS, resp.). Only lactose content was shown to differ significantly in these two groups. 

Lactose contributes the most to milk osmosis and it also belongs to the most unchanging (conserved) milk components. During mastitis, lactose production in damaged epithelial cells decreases, and it can be reabsorbed from milk into blood plasma [38]. Silanikove et al. [8] published results on how subclinical mastitis affects basic milk components and showed that only lactose decreased in milk with MCB, which was confirmed by our results as well. Leitner et al. [25] mention decreasing lactose content in MCB infected milk and conclude that lactose can be perused as an indicator of mammary gland inflammation. Leitner et al. [25] also found changes in other milk components due to subclinical mastitis, which is true neither for Silanikove et al. [8] nor for our results. As we have already mentioned, the results in our experiment are substantially influenced by low counts of (mostly coagulase-negative) *Staphylococcus* bacteria. Our results show that even a low count of coagulase-negative *Staphylococcus* bacteria nevertheless impacts milk quality. Many studies have confirmed that subclinical mastitis changes milk composition, which negatively influences nutritional and technological properties [8,39].

Our results confirmed our initial theory that both SCC and low counts of MCB impact goat milk quality. This realization made us divide the 34 samples into two groups (Table 6): LZ (with SCC <1000.10^3^/mL and *Staph.* count <100 CFU/mL) and all remaining samples (LBHZHB). The SCC value (<1000.10^3^/mL) in the LZ group above was chosen since (i) this SCC cut-off had the highest impact on milk quality (Table 4), (ii) milk quality is significantly influenced by *Staph.* count >100 CFU/mL MCB, and (iii) the distribution of *Staph.* spp. in groups L and H is even (Table 3).

The LBHZHB group had significantly higher protein, chloride, pH, and decreased lactose levels (Table 6). This group also tended toward an increased casein content. Most important, however, are the changes in technological parameters such as the lengthening of coagulation time, or shortened thermal stability, which might be caused by increased chloride content (as mentioned above) and/or pH change. A high pH in goat milk leads to increased curdling time and lower curd firmness [9,32]. It can be argued whether protein fragments originating from the activity of endogenous or exogenous enzymes in milk do also contribute to these observations. Higher casein content in those milk samples might cause the lengthening of coagulation time. As seen above, the division of samples into LZ vs. LBHZHB groups allowed us to reach clear conclusions about the impact on milk quality and technological properties. Goat farms should therefore screen routinely for these parameters to standardize and increase milk quality.

## 4. Conclusions

Somatic cell count (SCC) is still being neglected as a quality parameter in goat milk in the Czech Republic. In our study we were able to show the evidence for changes in milk quality with SCC > 600.10^3^/mL (5.58 LSCS) in a farm maintaining very high sanitation standards and without any significant presence of mastitis-causing bacteria (MCB) in the mammary gland. Generally speaking, an increasing SCC value in milk alters its technological properties. We argue that SCC is a neglected criterion that is able to impact dairy processing. Mastitis-causing bacteria in the mammary gland can alter milk quality, even when the microbial count is within the allowable limits for bulk milk mandated by the legislation. We hope that these results will lead to a better understanding of the problem and that they will increase both farmers’ knowledge and overall goat milk quality.

## Figures and Tables

**Table 1:** Microbiological, chemical, and technological parameters of goat milk samples according to lactation number.

Lactation Number	2nd		3rd		*p*
Samples (n)	12		22		
	Mean	SD	Mean	SD	
SCC (10^3^/mL) ^1^	1456	1215	1338	1009	0.857
LSCS ^2^	6.33	1.35	6.35	1.11	0.857
Milk yield (mL)	617	168	561	160	0.304
Rennetability (s)	155	60.5	146	33.1	0.885
Heat stability (s)	171	96.9	162	107	0.900
pH	6.84	0.12	6.81	0.09	0.322
log TMC (CFU/mL) ^3^	1.74	1.12	2.18	0.97	0.288
log *Staph.* (CFU/mL)	1.44	0.76	1.80	0.73	0.256
Fat (g/100 g)	5.07	0.78	5.66	1.27	0.220
Protein (g/100 g)	3.13	0.49	3.27	0.26	0.377
Casein (g/100 g)	2.53	0.44	2.62	0.30	0.368
Lactose (g/100 g)	4.60	0.17	4.54	0.17	0.272
Chlorides (mg/100 g)	157	10.06	155	13.90	0.540
NFDM (g/100 g) ^4^	8.55	0.50	8.58	0.29	0.746
TDM (g/100 g) ^5^	13.5	0.97	14.2	1.21	0.242

^1^ SCC—somatic cell count; ^2^ LSCS—linear somatic cell score; ^3^ TMC—total microbial count; ^4^ NFDM—non-fat dry matter; ^5^ TDM—total dry matter.

**Table 2:** Number of samples (n) according to SCC and MCB (*Staph.* count).

Group	*Staph.* Count (CFU/mL)	Samples (n)			
SCC (10^3^/mL) ^1^		<600	<700	<800 *	<1000
LSCS ^2^		<5.58	<5.81	<6.00	<6.32
LZ	<100	8	9	10	12
LB	>100	3	4	4	5
L	all	11	13	14	17
SCC (10^3^/mL) ^1^		>600	>700	>800 *	>1000
LSCS ^2^		>5.58	>5.81	>6.00	>6.32
HZ	<100	15	14	13	11
HB	>100	8	7	7	6
H	all	23	21	20	17
LBHZHB (∑ LB + HZ + HB)	all	-	-	-	22
Total samples (LZ + LB + HZ + HB)	all	34			
Z (=LZ + HZ)	<100	23			
B (=LB + HB)	>100	11			

* SCC cut-off 900 doesn’t exist because no investigated milk sample contained SCC 900–999.10^3^/mL. ^1^ SCC-somatic cell count; ^2^ LSCS-linear somatic cell score.

**Table 3:** Percentage (%) of MCB (positive samples with *Staph.* count >100 CFU/mL).

SCC Cut-Off (10^3^/mL) ^1^	600	700	800 *	1000
LSCS ^2^	5.58	5.81	6.00	6.32
LB out of L (SCC < cut-off)	27.3	30.8	28.6	29.4
HB out of H (SCC >cut-off)	34.8	33.3	35.0	35.3

* SCC cut-off 900 doesn’t exist because no investigated milk sample contained SCC 900–999.10^3^/mL. ^1^ SCC-somatic cell count; ^2^ LSCS-linear somatic cell score.

**Table 4:** Chemical and technological parameters in milk samples with *Staph.* count <100 CFU/mL. Twenty-three samples were subdivided into two groups with a lower (LZ) or higher (HZ) SCC than the particular SCC cut-off (600, 700, 800, or 1000.10^3^/mL).

Group	LZ		HZ		*p*	LZ		HZ		*p*	LZ		HZ		*p*	LZ		HZ		*p*
SCC (10^3^/mL) ^1^	<600		>600			<700		>700			<800 *		>800 *			<1000		>1000		
LSCS ^2^	<5.58		>5.58			<5.81		>5.81			<6.00		>6.00			<6.32		>6.32		
Samples (*n*)	8		15			9		14			10		13			12		11		
	Mean	SD	Mean	SD		Mean	SD	Mean	SD		Mean	SD	Mean	SD		Mean	SD	Mean	SD	
Milk yield (mL)	669 ^a^	136	510 ^b^	168	0.039	639	156	518	172	0.115	635	147	512	177	0.088	588	175	541	176	0.538
Rennetability (s)	129	29.0	159	54.2	0.087	131	28.0	160	56.2	0.176	131	26.4	163	57.7	0.129	129	25.4	171	59.0	0.053
Heat stability (s)	177	60.1	172	128	0.287	195	79.1	160	123	0.095	232 ^a^	137	129 ^b^	45.9	0.020	217 ^a^	129	126 ^b^	49.1	0.034
pH	6.79	0.08	6.82	0.12	0.540	6.81	0.09	6.81	0.12	0.875	6.80	0.09	6.82	0.12	0.515	6.78	0.09	6.84	0.12	0.091
Fat (g/100 g)	5.39	0.59	5.23	0.58	0.651	5.41	0.55	5.21	0.59	0.571	5.40	0.52	5.20	0.61	0.710	5.37	0.48	5.20	0.67	0.951
Protein (g/100 g)	3.09	0.17	3.29	0.42	0.071	3.05 ^b^	0.19	3.32 ^a^	0.41	0.017	3.08 ^b^	0.20	3.32 ^a^	0.42	0.035	3.04 ^b^	0.28	3.41 ^a^	0.35	0.009
Casein (g/100 g)	2.46	0.14	2.69	0.40	0.220	2.45	0.14	2.71	0.40	0.095	2.49	0.18	2.70	0.41	0.182	2.44 ^b^	0.23	2.80 ^a^	0.36	0.034
Lactose (g/100 g)	4.68	0.18	4.56	0.13	0.093	4.69 ^a^	0.17	4.55 ^b^	0.13	0.038	4.67	0.17	4.55	0.13	0.094	4.64	0.17	4.56	0.14	0.325
Chlorides (mg/100 mL)	145 ^b^	13.9	160 ^a^	12.2	0.024	146 ^b^	13.4	161 ^a^	12.5	0.027	146 ^b^	12.7	162 ^a^	12.4	0.013	147 ^b^	11.6	164 ^a^	12.3	0.004
NFDM ^3^ (g/100 g)	8.56	0.16	8.64	0.47	0.723	8.53	0.17	8.66	0.48	0.395	8.53	0.16	8.67	0.50	0.369	8.45 ^b^	0.28	8.79 ^a^	0.43	0.045
TDM ^4^ (g/100 g)	13.9	0.56	13.8	0.59	0.439	13.9	0.52	13.8	0.62	0.450	13.9	0.49	13.8	0.64	0.420	13.8	0.51	13.9	0.65	0.902

Statistical significance (*p* < 0.05) indicated by differing superscript letters (a, b). ^1^ SCC—somatic cell count; ^2^ LSCS—linear somatic cell score; ^3^ NFDM—non-fat dry matter; ^4^ TDM—total dry matter. * These two groups contain the same samples as they would for SCC 900.10^3^/mL.

**Table 5:** Chemical and technological parameters in milk samples according to MCB.

Group	Z		B		*p*
	*Staph.* spp. <100 CFU/mL		*Staph.* spp. >100 CFU/mL		
Samples (n)	23		11		
	Mean	SD	Mean	SD	
SCC (10^3^/mL) ^1^	1.471	1.209	1.188	706	0.956
LSCS ^2^	6.36	1.29	6.31	0.96	0.956
Milk yield (mL)	565	173	614	142	0.397
Rennetability (s)	149	48.6	149	34.9	0.659
Heat stability (s)	174	107	147	92.2	0.397
pH	6.81	0.11	6.84	0.07	0.418
Fat (g/100 g)	5.29	0.57	5.80	1.85	0.672
Protein (g/100 g)	3.22	0.36	3.22	0.37	0.868
Casein (g/100 g)	2.61	0.34	2.56	0.38	0.825
Lactose (g/100 g)	4.60 ^a^	0.16	4.47 ^b^	0.16	0.022
Chlorides (mg/100 mL)	155	14.6	157	6.92	0.619
NFDM (g/100 g)^3^	8.61	0.39	8.48	0.33	0.294
TDM (g/100 g)^4^	13.8	0.57	14.2	1.90	0.727

Statistical significance (*p* < 0.05) indicated by differing superscript letters (a, b). Z group includes samples from LZ + HZ groups (Table 2), whereas B group includes samples from LB + HB groups (Table 2). ^1^ SCC-somatic cell count; ^2^ LSCS-linear somatic cell score; ^3^ NFDM-non-fat dry matter, ^4^ TDM-total dry matter.

**Table 6:** Chemical and technological parameters in milk samples according to SCC and MCB.

Group	LZ		LBHZHB		*p*
SCC (10^3^/mL) ^1^	<1000		*		
LSCS ^2^	<6.32		*		
*Staph.* Count (CFU/mL)	<100		any		
Samples (*n*)	12		22		
	Mean	SD	Mean	SD	
Milk yield (mL)	588	175	577	160	0.857
Rennetability (s)	129 ^b^	25.4	160 ^a^	48.6	0.047
Heat stability (s)	217 ^a^	129	137 ^b^	72.9	0.031
pH	6.78 ^b^	0.09	6.84 ^a^	0.09	0.045
Fat (g/100 g)	5.37	0.48	5.50	1.39	0.857
Protein (g/100 g)	3.04 ^b^	0.28	3.31 ^a^	0.37	0.031
Casein (g/100 g)	2.44	0.23	2.68	0.38	0.066
Lactose (g/100 g)	4.64 ^a^	0.17	4.52 ^b^	0.15	0.050
Chlorides (mg/100 mL)	147 ^b^	11.6	160 ^a^	10.4	0.002
NFDM (g/100 g) ^3^	8.45	0.28	8.63	0.40	0.296
TDM (g/100 g) ^4^	13.8	0.51	14.0	1.40	0.719

Statistical significance (*p* < 0.05) indicated by differing superscript letters (a, b). * See Table 2. ^1^ SCC-somatic cell count; ^2^ LSCS-linear somatic cell score; ^3^ NFDM-non-fat dry matter; ^4^ TDM-total dry matter.

## Data Availability

The data presented in this study are available on request from the corresponding author.
